# Supplementary material for: A novel lipophilic amiloride derivative efficiently kills chemoresistant breast cancer cells
Source: Sci Rep. 2024 Aug 31;14:20263. doi: 10.1038/s41598-024-71181-0 (PMC11365969; doi:10.1038/s41598-024-71181-0)
Supplement: Supplementary file 1 — Supplementary Information. [file 41598_2024_71181_MOESM1_ESM.pdf]

Supplementary Materials

Structure-Activity Relationship Study Identifies a Novel Lipophilic Amiloride Derivative that Efficiently Kills Chemoresistant Breast Cancer Cells

Michelle Hu, Ruiwu Liu, Noemi Castro, Liliana Loza-Sanchez, Lapamas Rueankham, Julie A. Learn, Ruiqi Huang, Kit S. Lam, and Kermit L. Carraway, III*

*** Correspondence:** klcarraway@ucdavis.edu

Cell Line	Species	Subtype	ER	PR	HER2	Tissue type	Source
MDA-MB-231	Human	Basal	-	-	-	Metastatic Adenocarcinoma	ATCC #HTB-26
MCF7	Human	Luminal	+	+	-	Metastatic Adenocarcinoma	ATCC #HTB-22
T47D	Human	Luminal	+	+	-	Invasive Ductal Carcinoma	ATCC #HTB-133
SKBR3	Human	Luminal	-	-	+	Adenocarcinoma	ATCC #HTB-30
MCF7 TS	Human	Luminal	+	+	-	Metastatic Adenocarcinoma	Gifted by ATCC PBCF
MCF7 TR-1	Human	Luminal	+	+	-	Metastatic Adenocarcinoma	Gifted by ATCC PBCF
MCF7 TR-5	Human	Luminal	+	+	-	Metastatic Adenocarcinoma	Gifted by ATCC PBCF
MCF7 MX-100	Human	Luminal	+	+	-	Metastatic Adenocarcinoma	Gifted by A.M Yu
4T1	Mouse	Luminal, basal	-	-	-	Metastatic Adenocarcinoma	ATCC #CRL-2539
Met-1	Mouse	Luminal	-	unk	unk	Metastatic Adenocarcinoma	Gifted by A. Borowsky
NDL	Mouse	Luminal	-	-	+	Invasive Ductal Carcinoma	Derived from primary tumor
UCDK9 MM3	Dog	unk	unk	unk	unk	Melanoma	Gifted by R. Rebhun
UCDK9 OSA29	Dog	unk	unk	unk	unk	Osteosarcoma	Gifted by R. Rebhun
D17	Dog	unk	unk	unk	unk	Osteosarcoma	Gifted by R. Rebhun

Supplementary Table S1. Sources and characteristics of cancer cell lines employed in this study. Cell lines from human, mouse and dog origins were previously derived from the indicated tumor tissue types and classified based on cell of origin (basal or luminal) and hormone receptor expression status (estrogen receptor (ER), progesterone receptor (PR), and epidermal growth factor receptor 2/human epidermal growth factor receptor 2 (HER2). Receptor overexpression is shown as positive (+), negative (-) or unknown (unk). Several cell lines were purchased from American Type Culture Collection (ATCC).

Compound	Final Concentration	Solvent	Source
10357	0-500 μM	DMSO	Gifted by F.A. Gorin
Amiloride	0-500 μM	DMSO	#A7410, Sigma Aldrich
Benzamil	0-500 μM	DMSO	#B2417, Sigma Aldrich
HMA	0-100 μM	DMSO	#A9561, Sigma Aldrich
LLC1	0-100 μM	DMSO	Synthesized by CCCBSR
LLC2	0-100 μM	DMSO	Synthesized by CCCBSR
LLC3	0-100 μM	DMSO	Synthesized by CCCBSR
LLC4	0-100 μM	DMSO	Synthesized by CCCBSR
LLC5	0-100 μM	DMSO	Synthesized by CCCBSR
LLC7	0-100 μM	DMSO	Synthesized by CCCBSR
LLC8	0-100 μM	DMSO	Synthesized by CCCBSR
LLC9	0-100 μM	DMSO	Synthesized by CCCBSR
LLC10	0-500 μM	DMSO	Synthesized by CCCBSR
Docetaxel	50nM – 10μM	DMSO	#S1148, Selleckchem
Doxorubicin	75nM – 10μM	H_2O	#BP25165, Fisher Scientific
(Z)-4-Hydroxytamoxifen	250nM – 10μM	EtOH	#H7904, Sigma Aldrich

Supplementary Table S2. Characteristics of drugs employed in this study. The final concentrations, solvents, and sources of all agents are summarized. Abbreviations used: dimethylsulfoxide (DMSO), ethanol (EtOH). LLC1 through LLC10 were synthesized, purified and characterized by the Combinatorial Chemistry and Chemical Biology Shared Resource (CCCBSR) of the UC Davis Comprehensive Cancer Center.

Murine	HMA (μM)	LLC1 (μM)	Canine	HMA (μM)	LLC1 (μM)
Met-1	24 ± 4	7 ± 0.1	UCDK9MM3	35 ± 9	11 ± 2
NDL	29 ± 6	8 ± 0.6	UCDK9OSA29	25 ± 4	12 ± 2
4T1	24 ± 2	9 ± 1.6	D17	42 ± 2	14 ± 1

Supplementary Table S3. HMA and LLC1 are effective toward murine mammary tumor and canine cancer cell lines. Several murine and canine cell lines were titrated with LLC1 or HMA for 24hr to determine viability via MTT assay. IC_{50} values are shown in micromolar. Data represent three biological replicates.

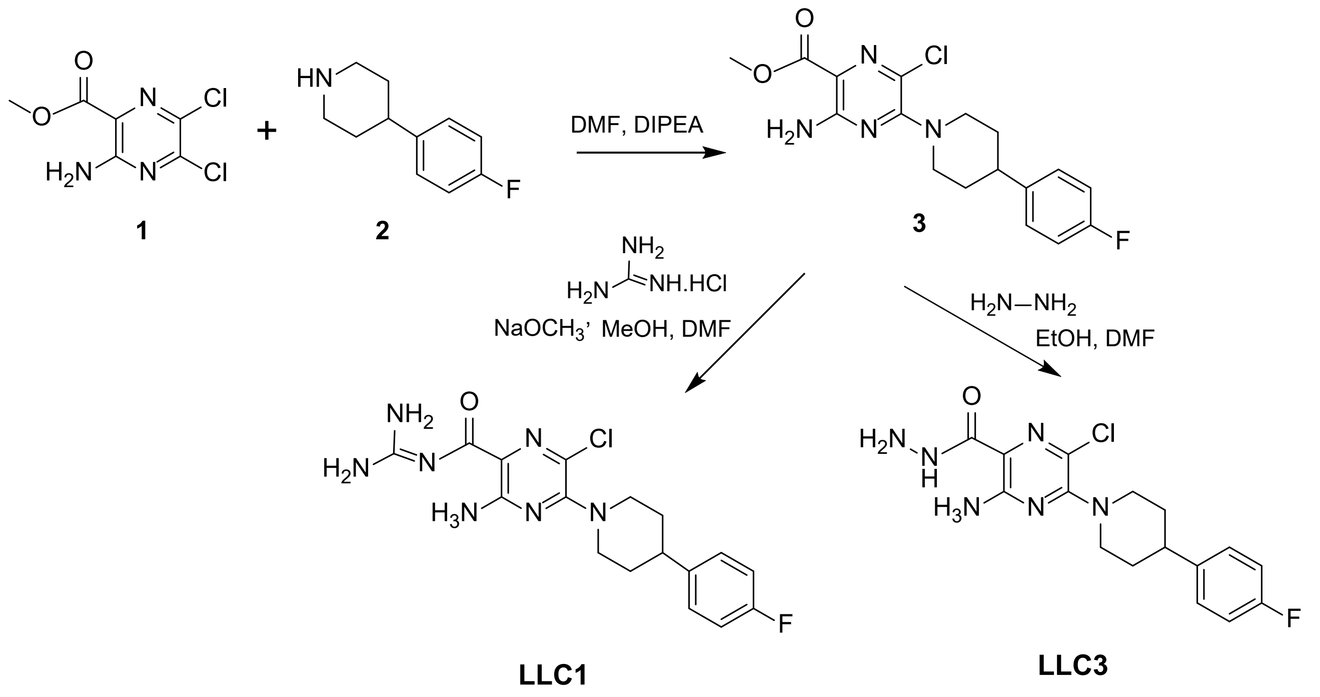

Supplementary Figure S1. Synthetic scheme for LLC1 and LLC3.

A

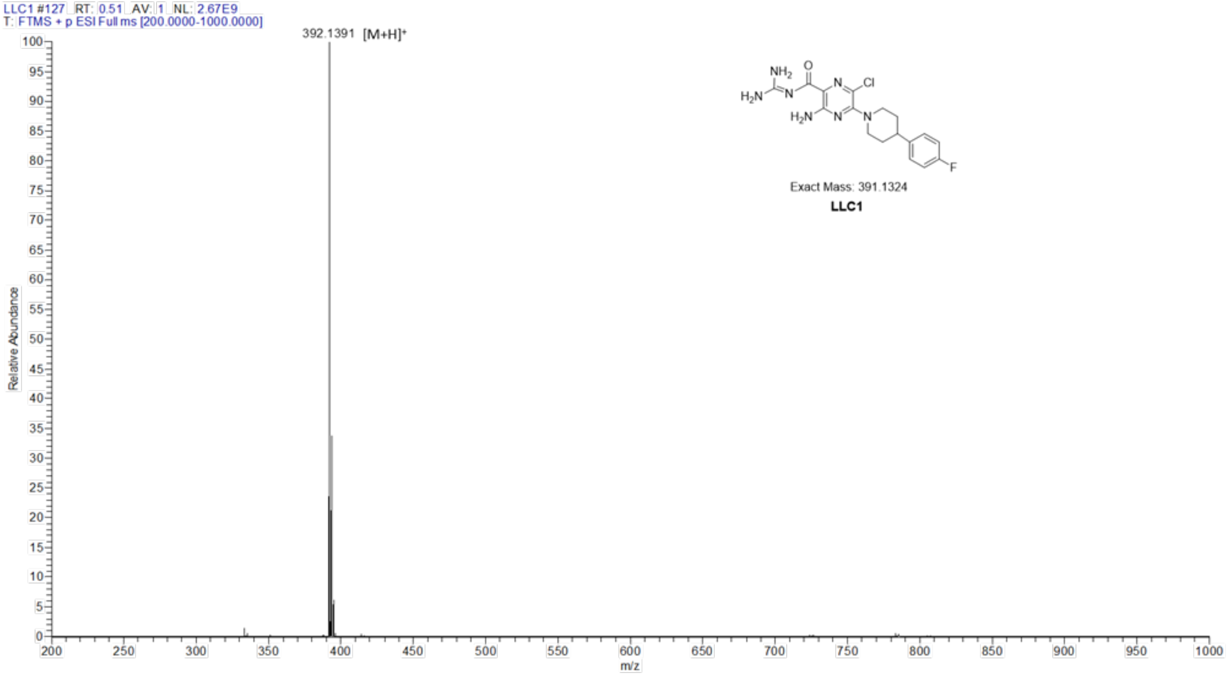

B

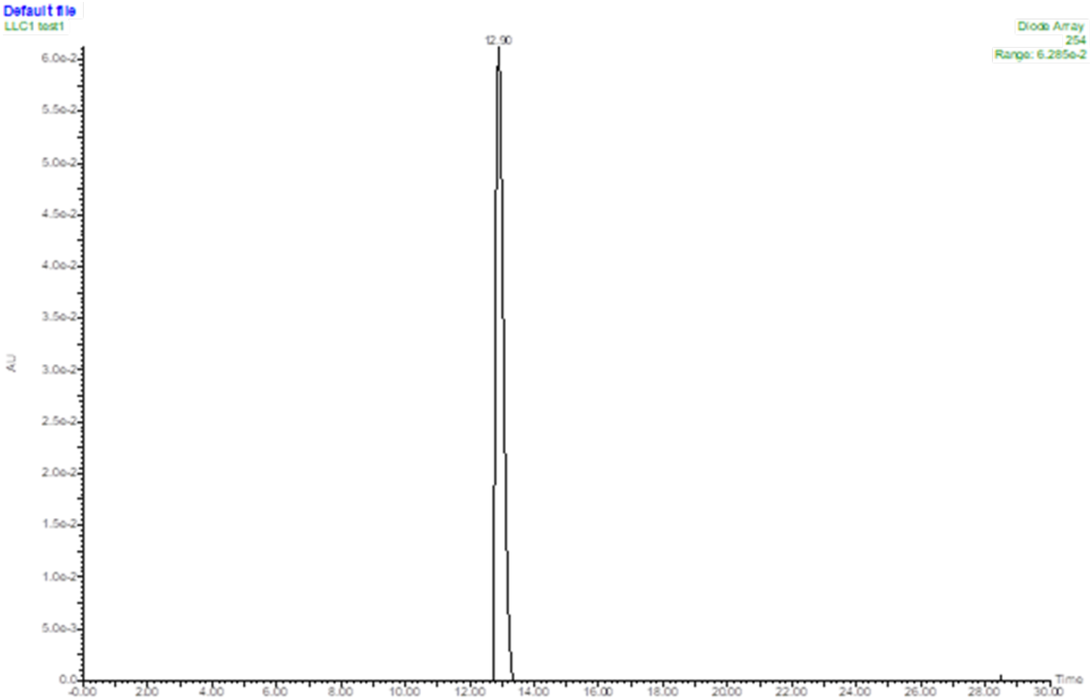

Supplementary Figure S2. (A) ESI-MS of LLC1. ESI-MS confirmed the structure of LLC1: calculated: m/z 392.1402, found: 392.1391 $[M+H]^+$. **(B)** HPLC of LLC1. HPLC chromatogram showed purity of LLC1 was > 98%**.**

A

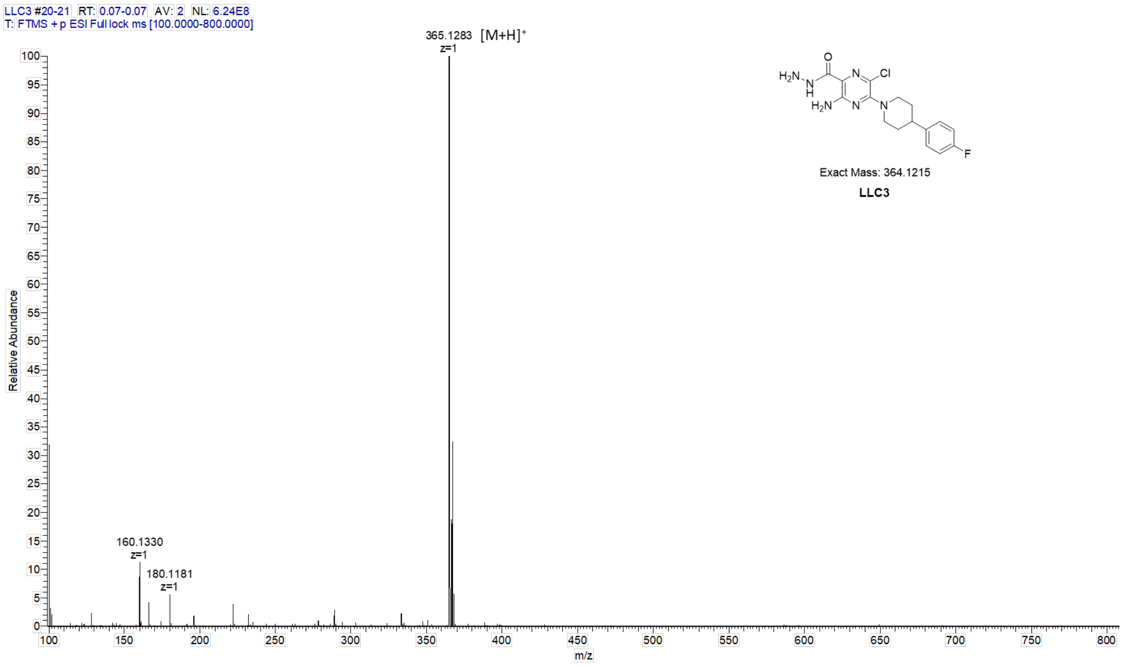

B

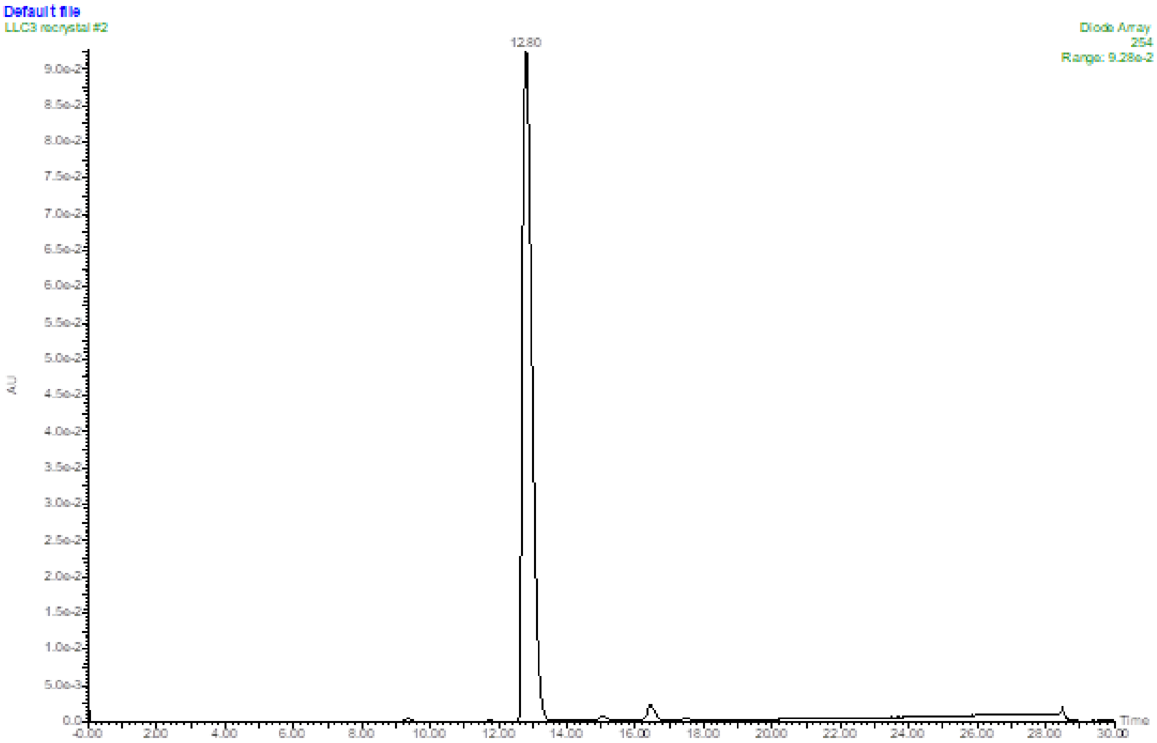

Supplementary Figure S3. (A) ESI-MS of LLC3. ESI-MS confirmed the structure of LLC3: calculated: m/z 365.1293, found: 392.1283 $[M+H]^+$. **(B)** HPLC of LLC3.

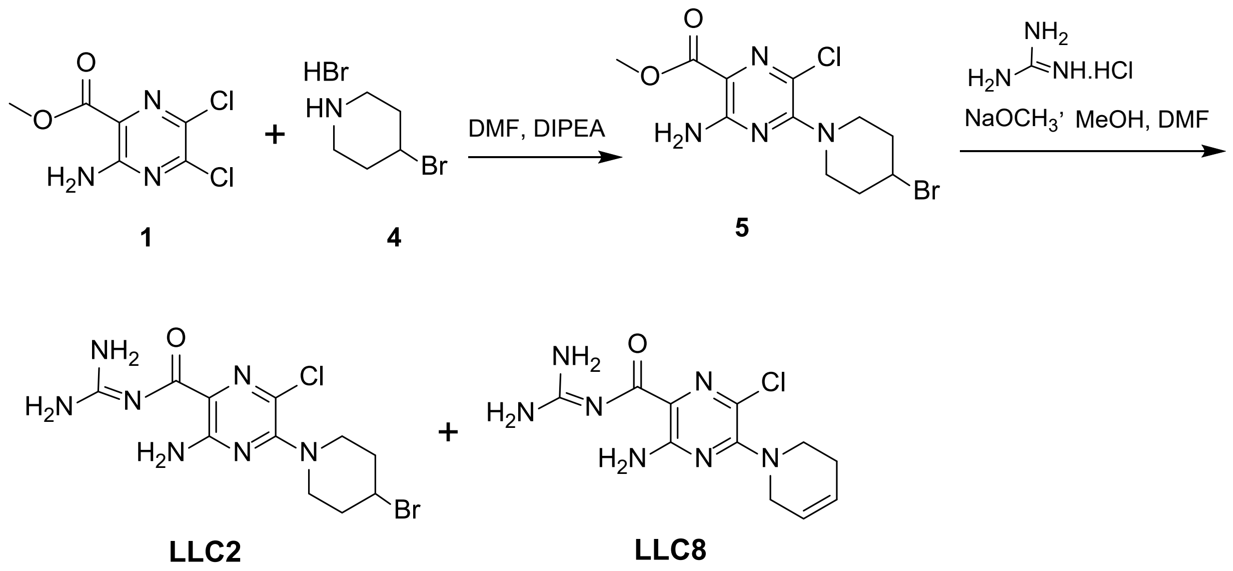

Supplementary Figure S4. Synthetic scheme for LLC2 and LLC8.

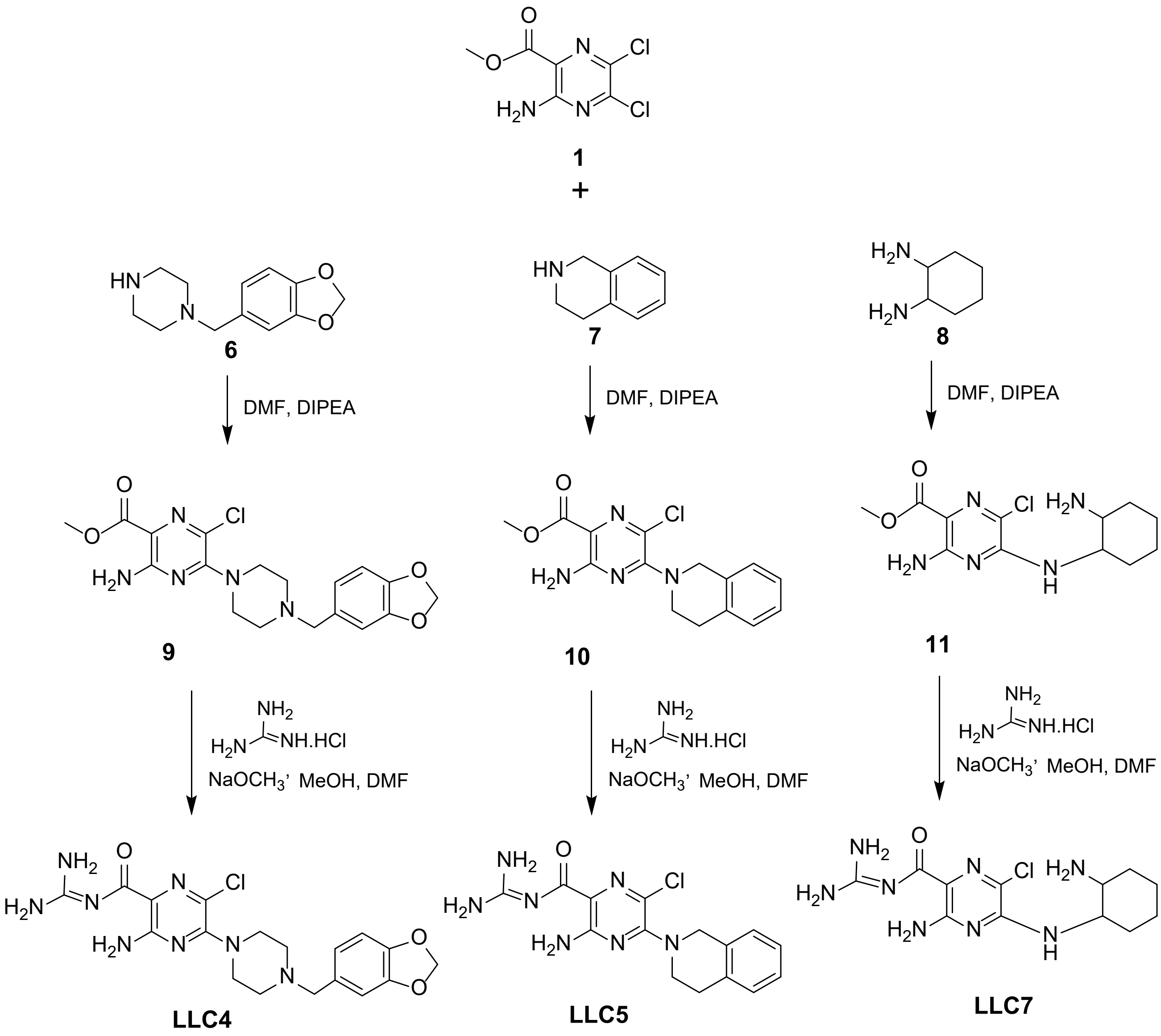

Supplementary Figure S5. Synthetic schemes for LLC4, LLC5 and LLC7.

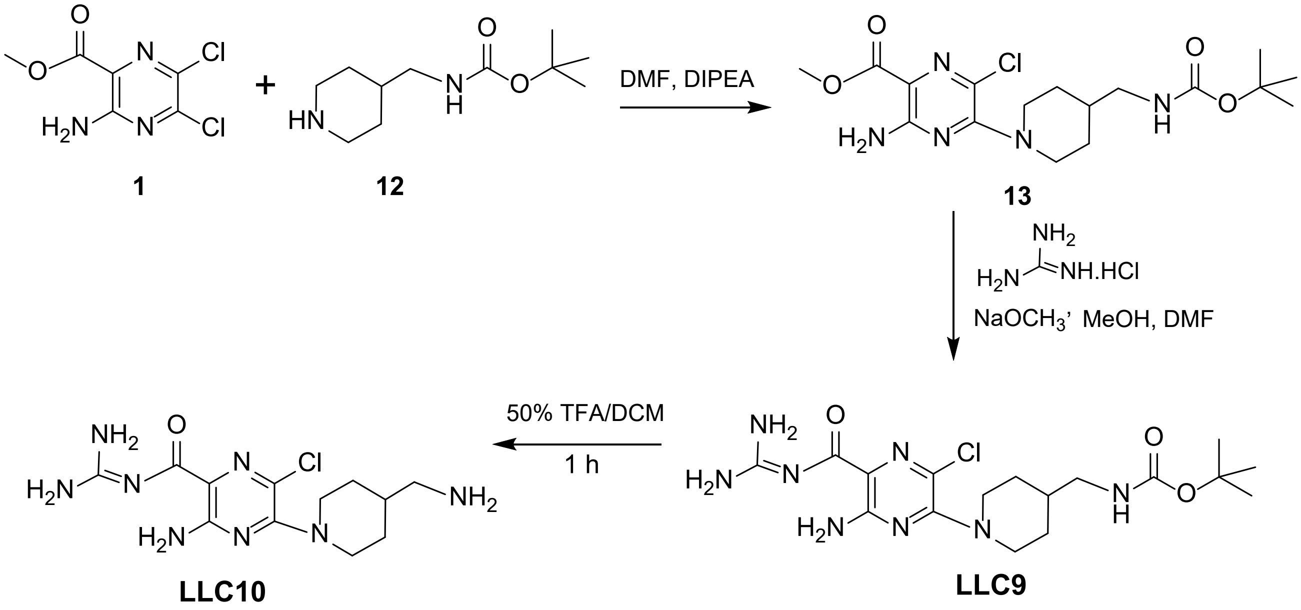

Supplementary Figure S6. Synthetic schemes for LLC9 and LLC10.

A

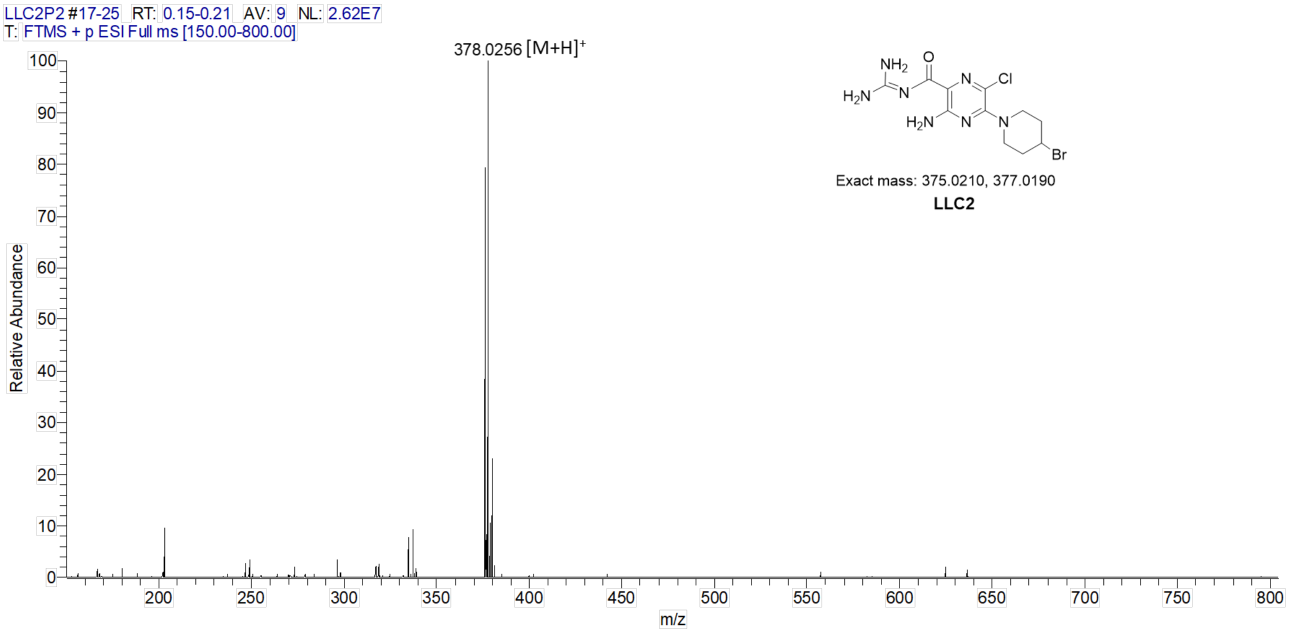

B

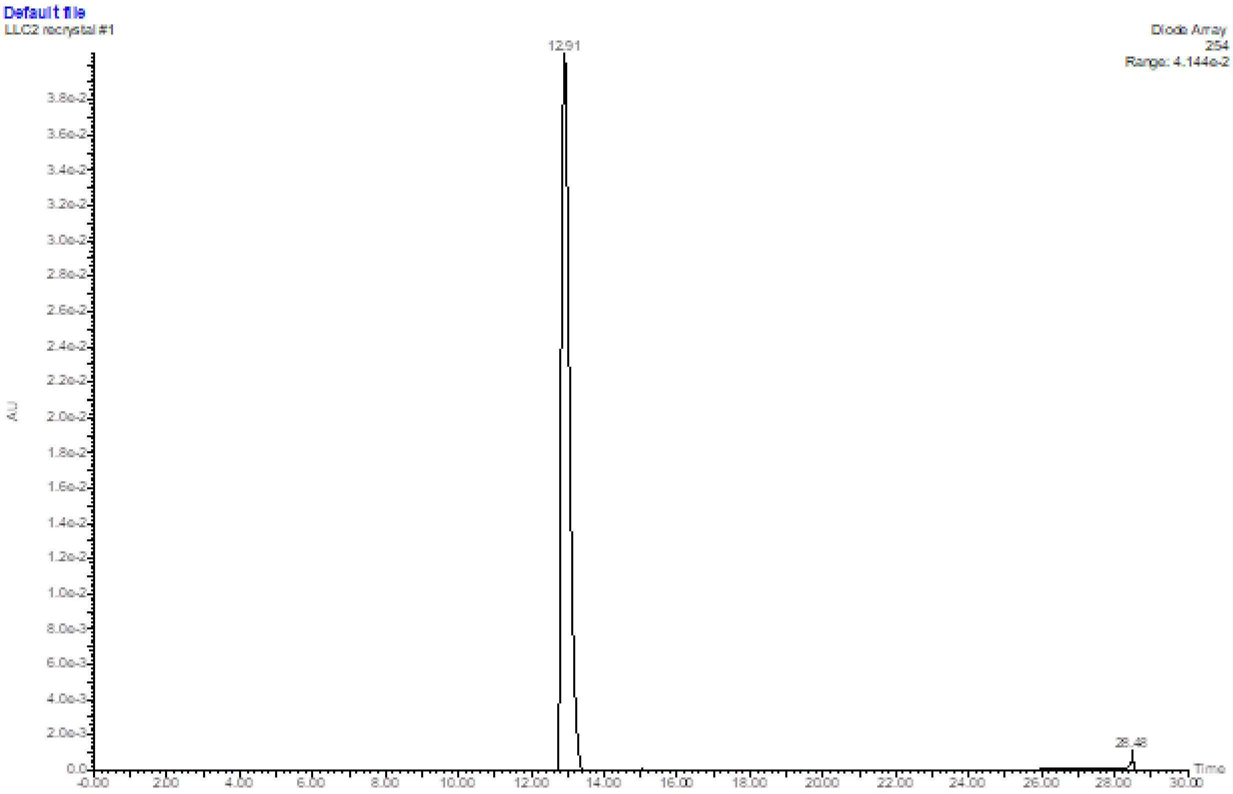

Supplementary Figure S7. (A) ESI-MS of LLC2. ESI-MS confirmed the structure of LLC2: calculated: m/z 376.0288, 378.0268, found: 376.0274, 378.0256 $[M+H]^+$. **(B)** HPLC of LLC2.

A

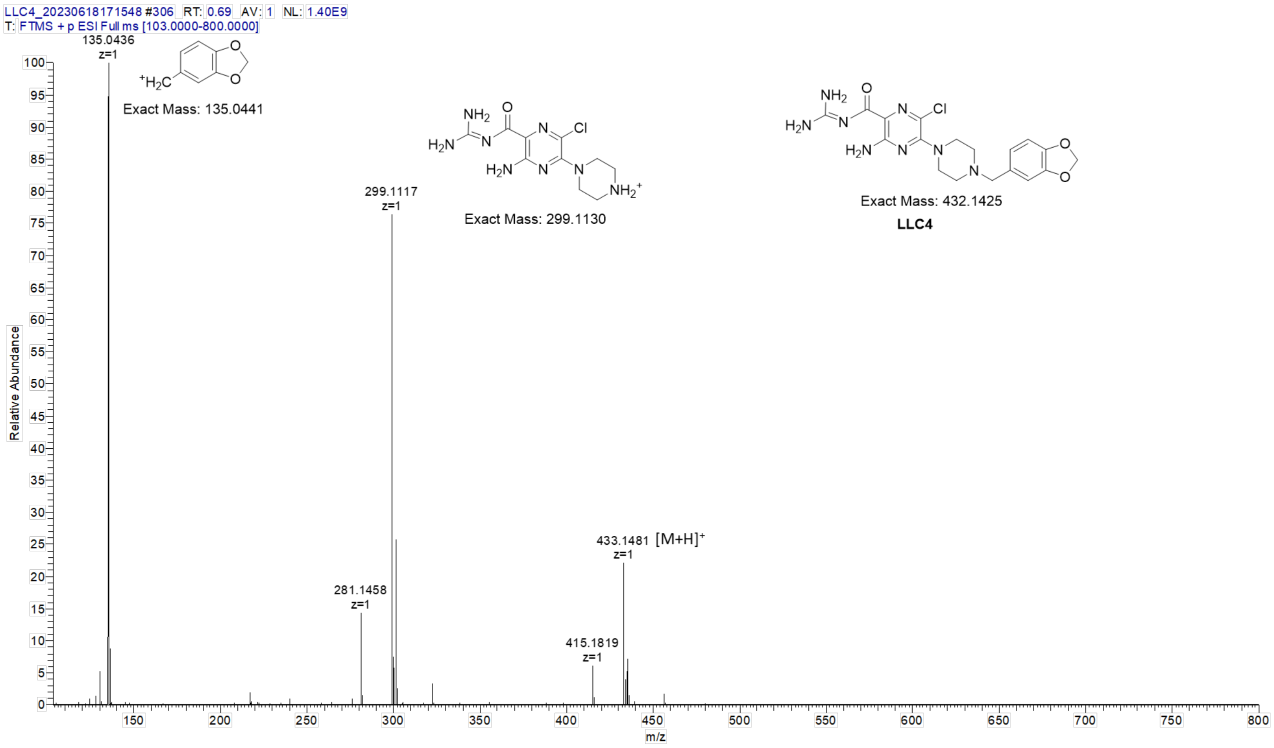

B

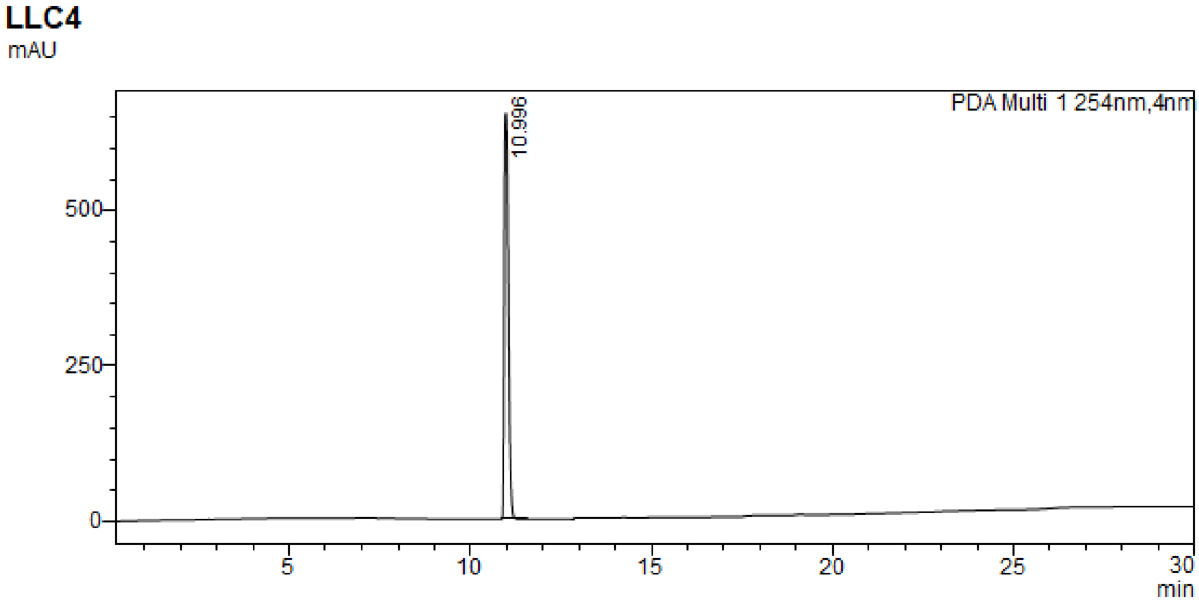

Supplementary Figure S8. (A) ESI-MS of LLC4. ESI-MS confirmed the structure of LLC4: calculated: m/z 433.1503, found: 433.1481 $[M+H]^+$, fragment peaks: calculated: m/z 135.0441, found: 135.0436; calculated: m/z 299.1130, found: 299.1117. **(B)** HPLC of LLC4.

A

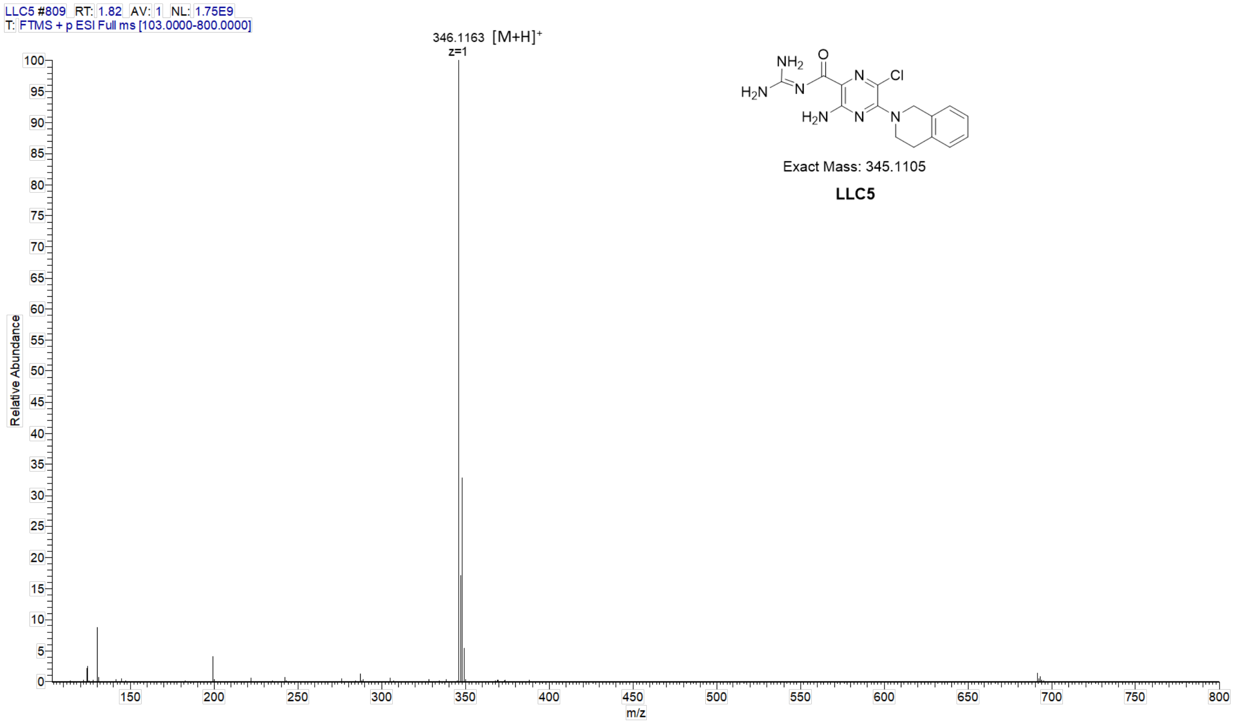

B

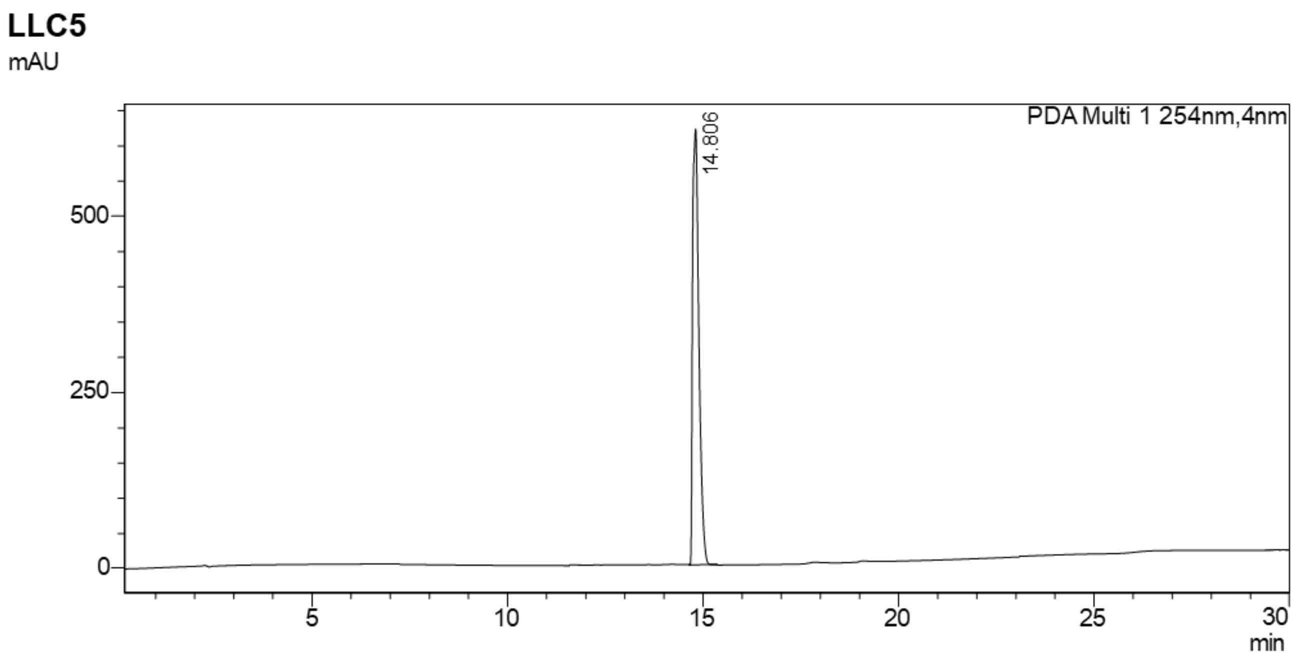

Supplementary Figure S9. (A) ESI-MS of LLC5. ESI-MS confirmed the structure of LLC5: calculated: m/z 346.1183, found: 346.1163 $[M+H]^+$. **(B)** HPLC of LLC5.

A

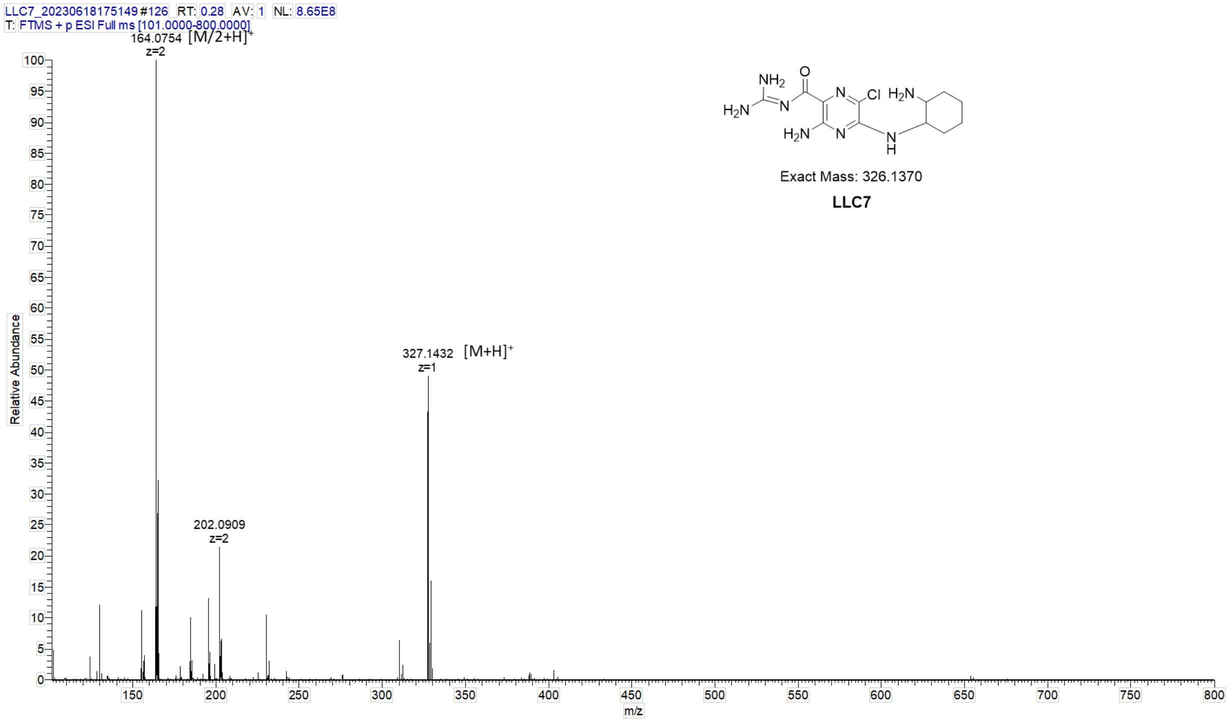

B

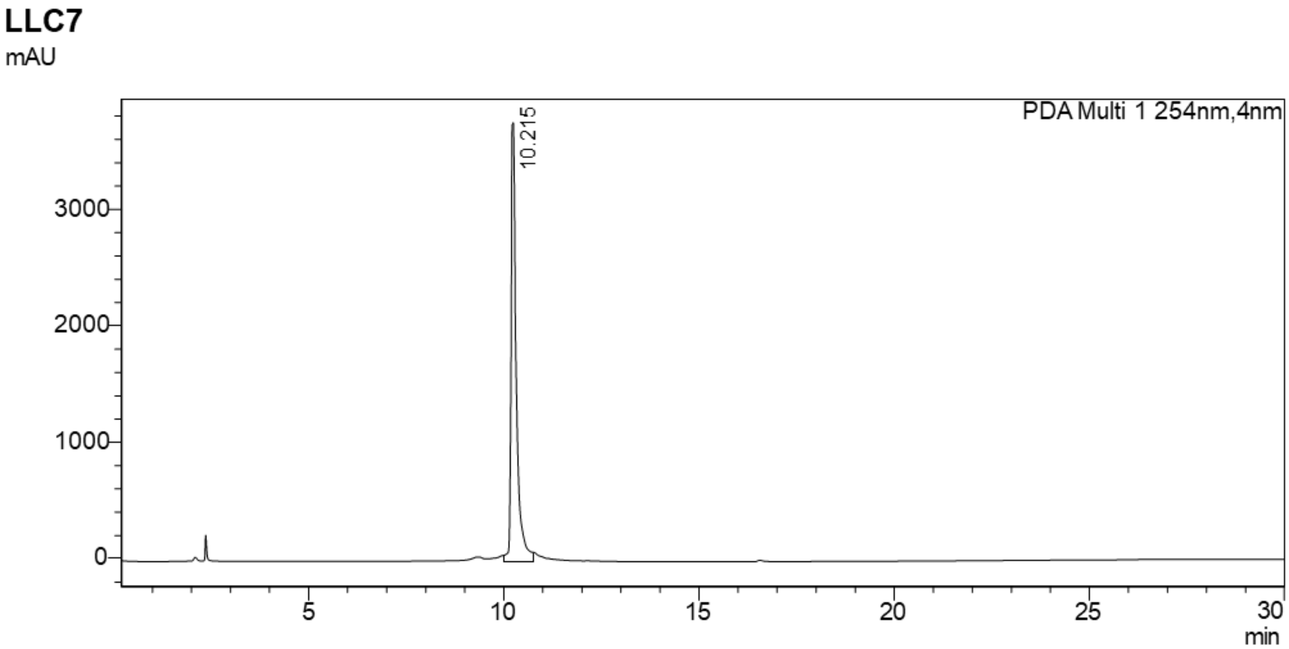

Supplementary Figure S10. (A) ESI-MS of LLC7. ESI-MS confirmed the structure of LLC7: calculated: m/z 327.1449, found: 327.1432 [M+H]$^+$, 164.0754 [M/2+H]$^+$. **(B)** HPLC of LLC7.

A

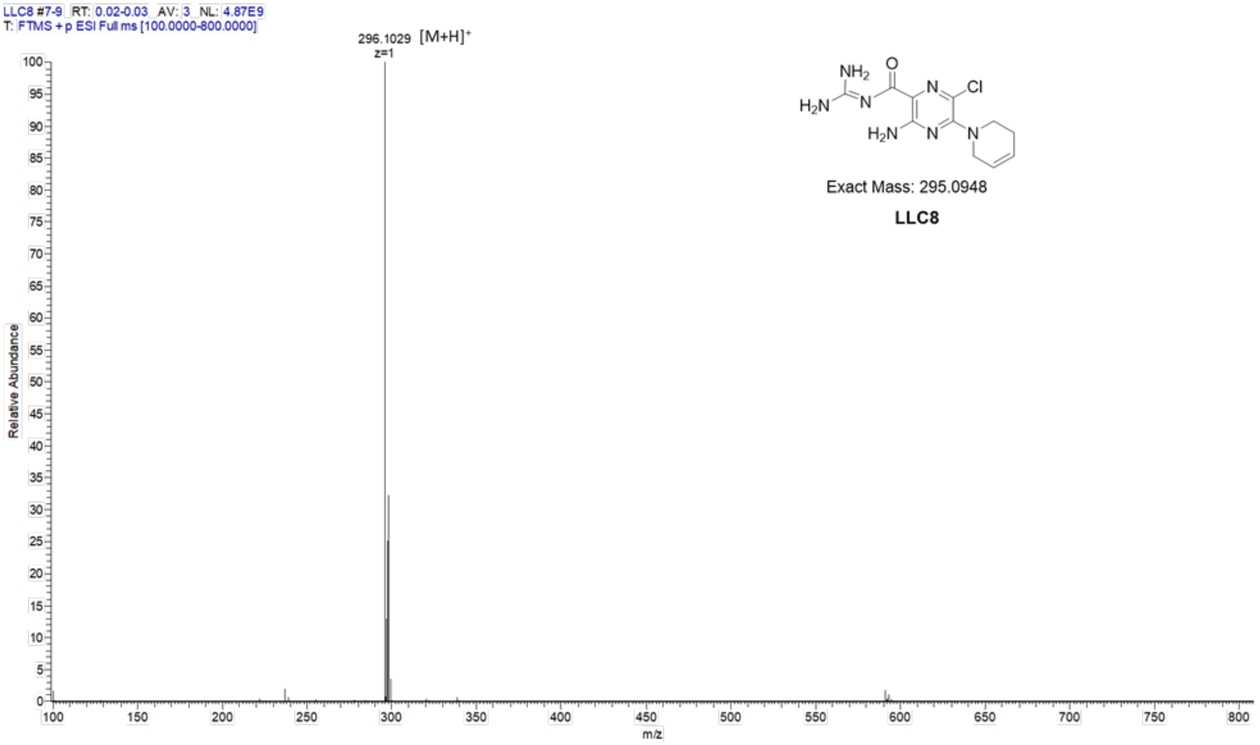

B

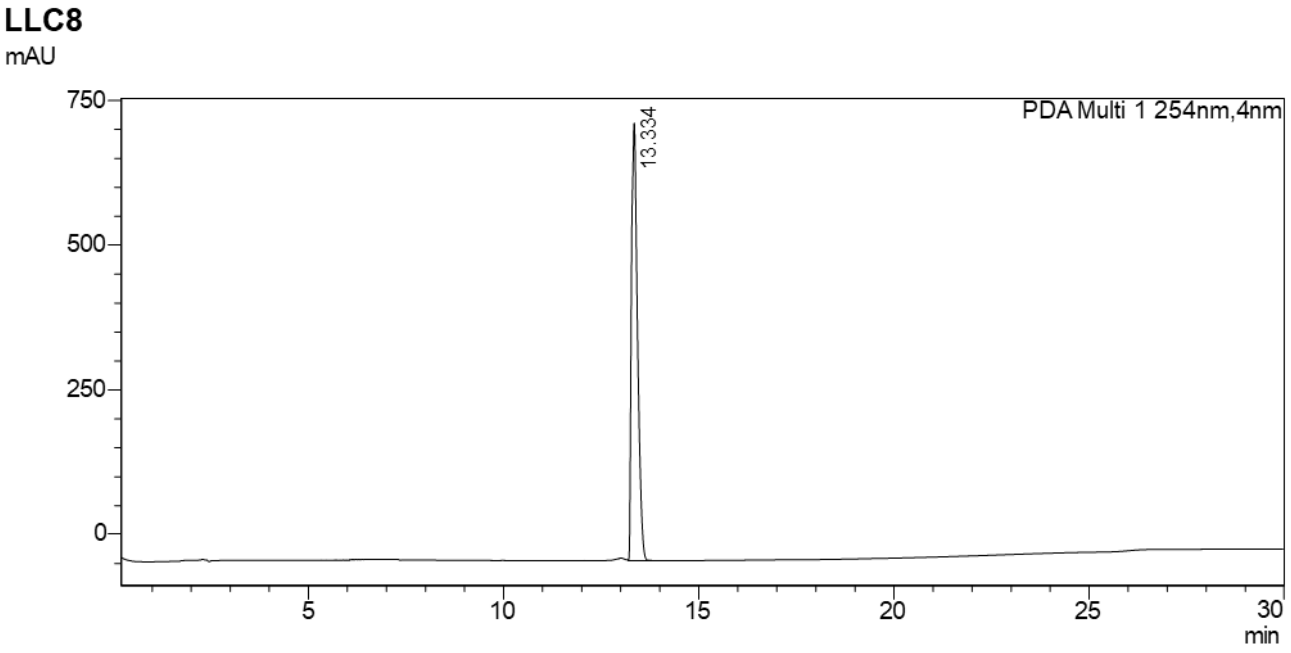

Supplementary Figure S11. (A) ESI-MS of LLC8. ESI-MS confirmed the structure of LLC8: calculated: m/z 296.1027, found: 296.1029 $[M+H]^+$. **(B)** HPLC of LLC8.

A

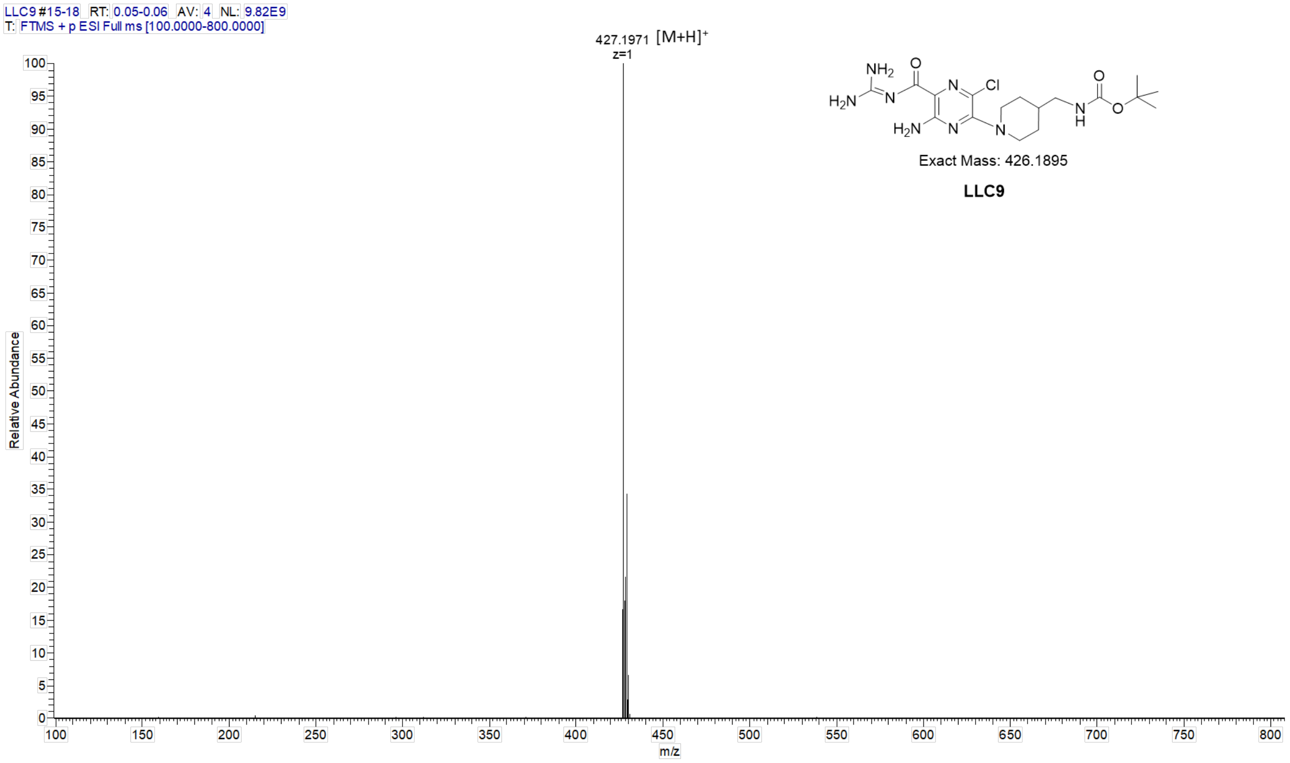

B

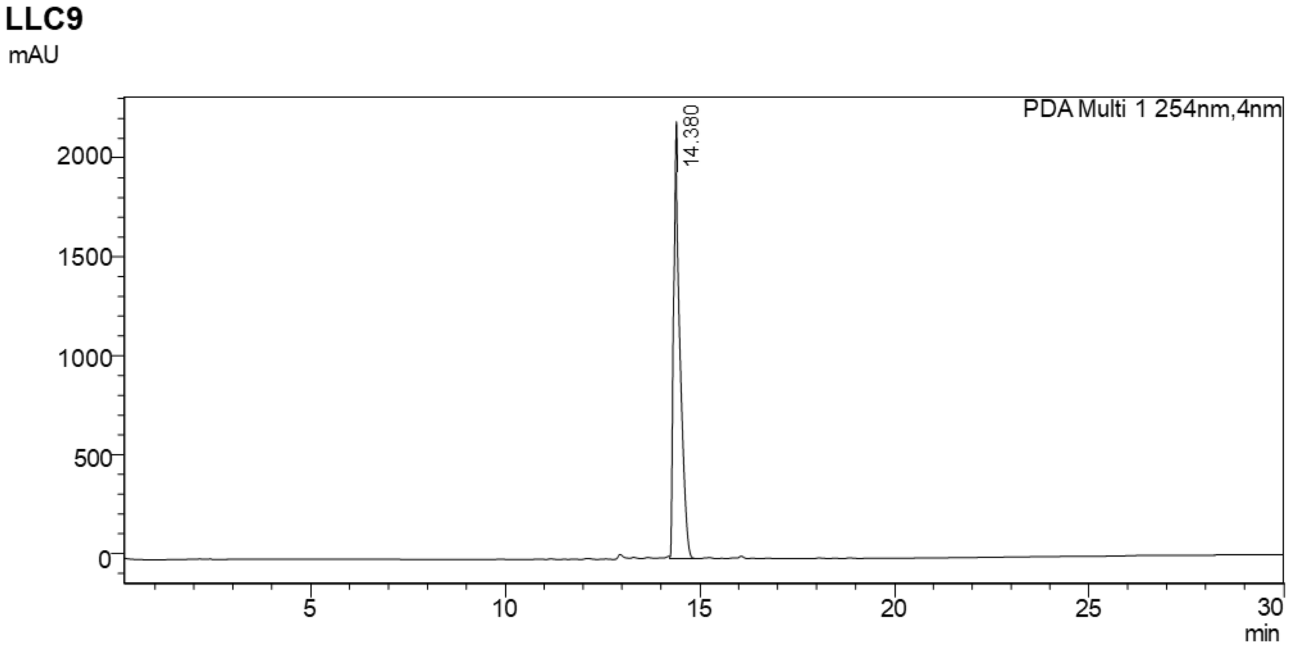

Supplementary Figure S12. (A) ESI-MS of LLC9. ESI-MS confirmed the structure of LLC9: calculated: m/z 427.1973, found: 427.1971 $[M+H]^+$. **(B)** HPLC of LLC9.

A

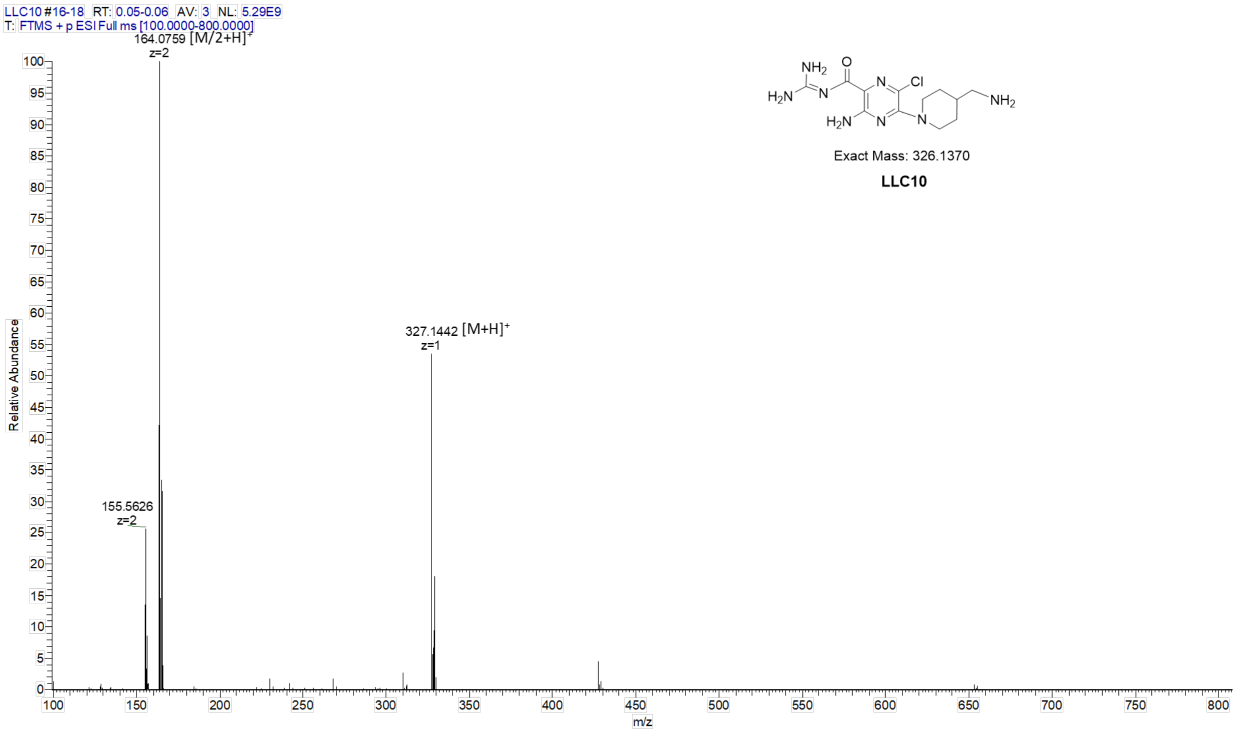

B

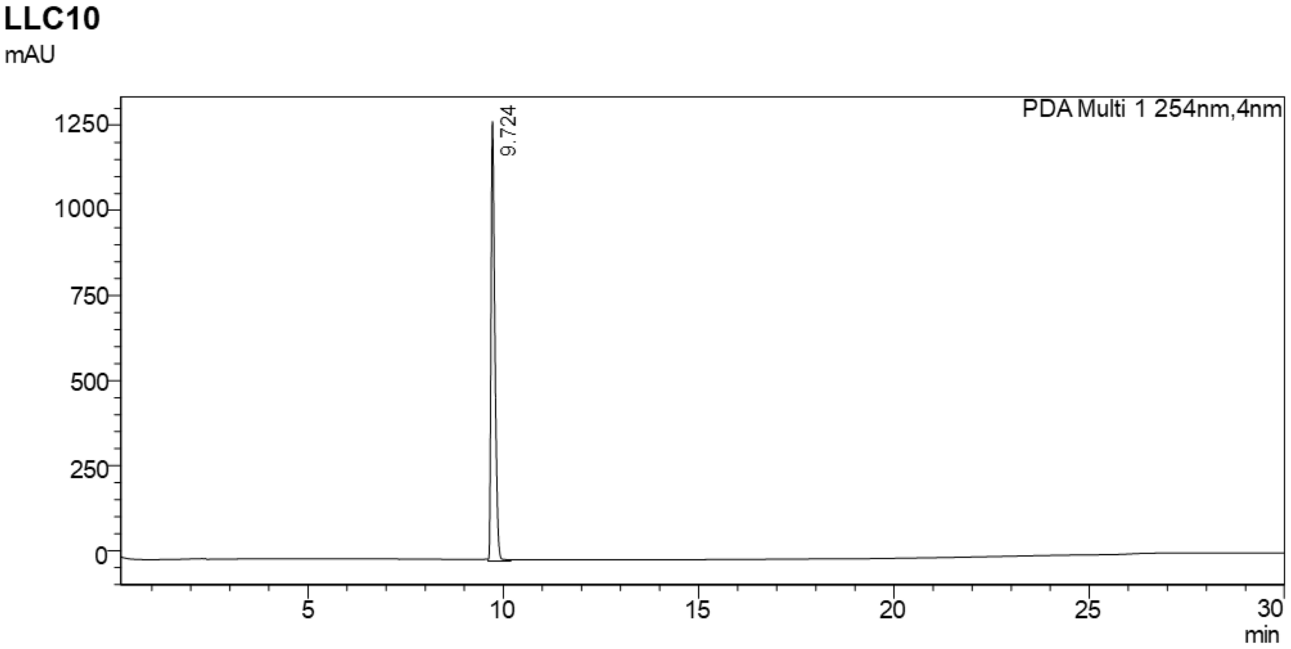

Supplementary Figure S13. (A) ESI-MS of LLC10. ESI-MS confirmed the structure of LLC10: calculated: m/z 327.1449, found: 327.1442 $[M+H]^+$. 164.0759 $[M/2+H]^+$. **(B)** HPLC of LLC10.

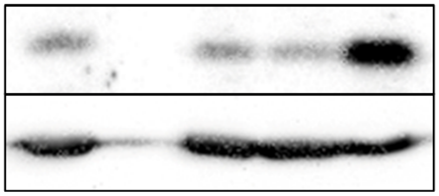

Supplementary Figure S14. LLC1- and HMA do not provoke apoptotic cell death. MDA-MB-231 cells were treated for 24hr as indicated with DMSO, 40 μM HMA, or 10 μM LLC1, or for 72hr with 10 μM doxorubicin as a positive control for drug-induced apoptosis. Lysates were immunoblotted with antibodies for cleaved caspase 7 (Cell Signaling, 9491) and tubulin (Sigma, T5168).

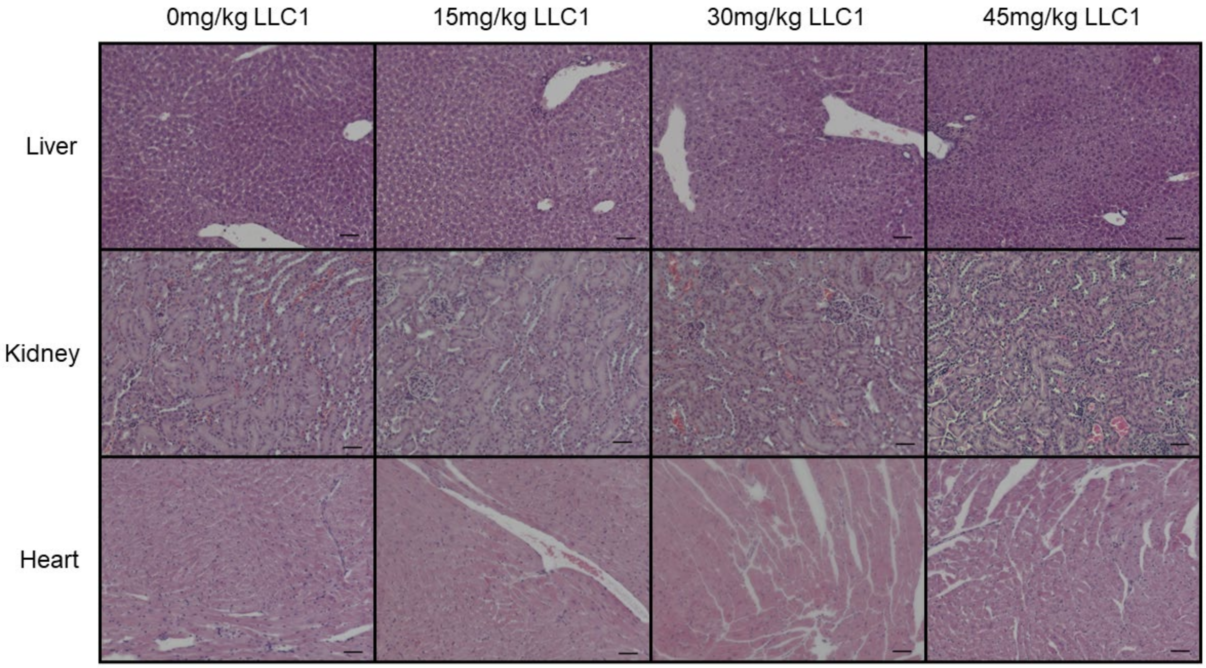

Supplementary Figure S15. Histopathology of tissues after LLC1 treatment. H&E staining of 5μm paraffin-embedded tissue sections of liver, kidney, and heart from the maximum tolerated dose experiment is depicted (scale bar = 50μm).

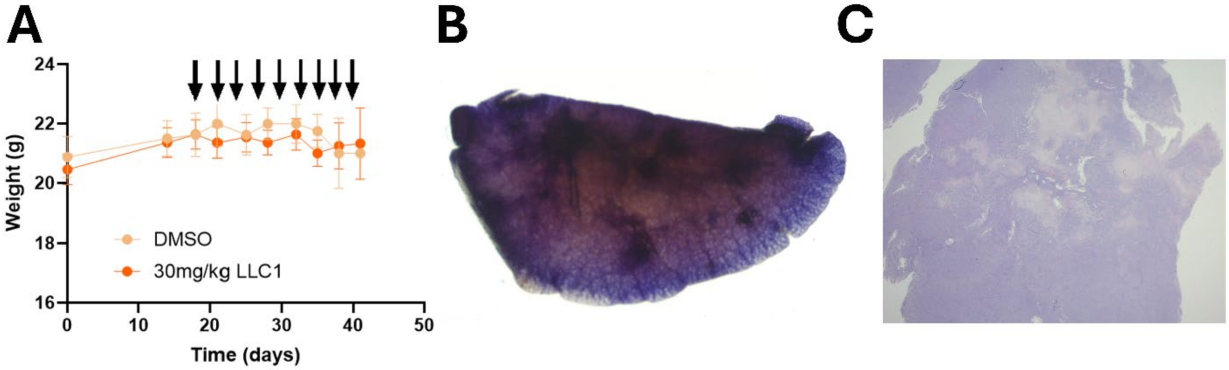

Supplementary Figure S16. Supporting data for mouse therapeutic study. (A) Average animal body weights (±SEM) over the course of drug treatment were plotted. Arrows depict treatment days. **(B)** Representative whole mount image of a lung lobe from a vehicle-treated mouse. This lobe was scored as containing eight metastatic lesions. **(C)** Representative H&E staining of tumor tissue from an LLC1-treated mouse. Areas of pink or gray were scored as necrotic.

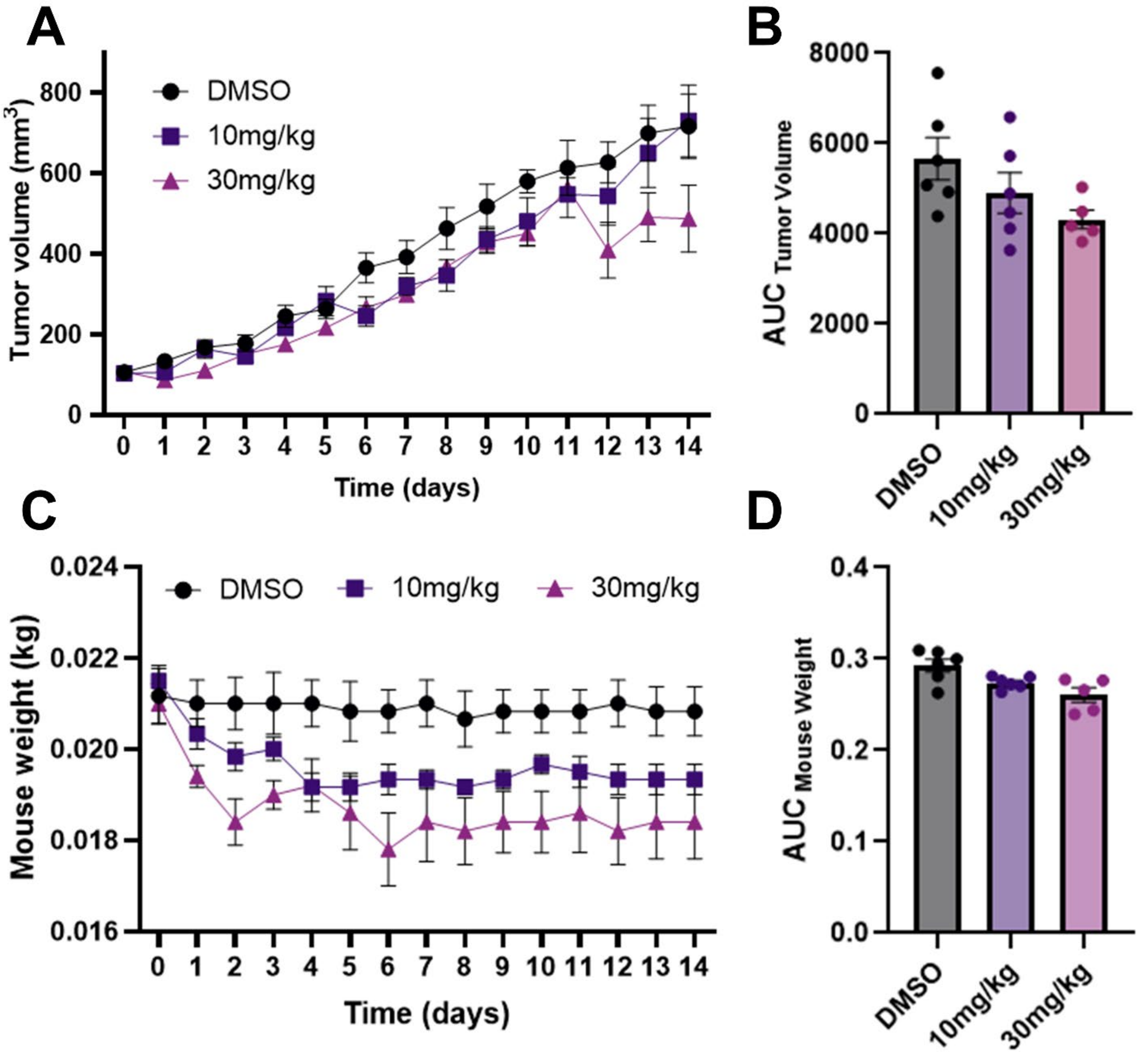

Supplementary Figure S17. Aggressive treatments of mice with LLC1. Animals were treated twice daily for two weeks with vehicle (n=6), 10mg/kg LLC1 (n=6) or 30mg/kg LLC1 (n=5). **(A)** Average tumor volumes are plotted for each group versus time. **(B)** The integrated areas under the tumor volume curve ($AUC_{\text{Tumor Volume}}$) were calculated using GraphPad. Statistical significance was calculated using an unpaired two-tailed t-test or ordinary one-way ANOVA with multiple comparisons. DMSO vs 10mg/kg: t-test: P = 0.2686, ANOVA: P = 0.3621; DMSO vs 30mg/kg: t-test: P = 0.0380, ANOVA: P = 0.0781. **(C)** Average mouse weights for each group are plotted versus time. **(D)** The integrated areas under the weight curve ($AUC_{\text{Mouse Weight}}$) were calculated using GraphPad. Statistical significance was calculated using an unpaired two-tailed t-test or ordinary one-way ANOVA with multiple comparisons. DMSO vs 10mg/kg: t-test: P = 0.0354, ANOVA: P = 0.0894; DMSO vs 30mg/kg: t-test: P = 0.0160, ANOVA: P = 0.0062. Error bars in all panels represent SEM.
